# A randomized, double-blind, placebo controlled, parallel group, efficacy study of alpha BRAIN^® ^administered orally

**DOI:** 10.1186/1550-2783-12-S1-P54

**Published:** 2015-09-21

**Authors:** Todd M Solomon, Jarrett Leech, Cynthia Murphy, Guy DeBros, Andrew Budson, Paul Solomon

**Affiliations:** 1 Boston Center for Memory, Newton, MA, USA; 2 Onnit Labs, Austin, TX, USA; 3 Memory Clinic, Bennington, VT, USA; 4 Boston University School of Medicine, Boston, MA, USA; 5 Williams College, Williamstown, MA, USA

## Background

Nutritional supplements that purport cognitive enhancing properties are widely available and are being consumed by athletes with increasing prevalence. The goal of this study was to investigate the efficacy of a self-described cognitive enhancing nutraceutical on cognitive functioning in a group of healthy adults by utilizing a randomized, double-blind, placebo controlled design.

## Methods

A total of 63-treatment naïve individuals participated in this randomized, double-blind, placebo controlled trial. All participants completed a two-week placebo run in before receiving either active product, Alpha BRAIN^® ^or new placebo. Participants then followed the manufactures recommended instructions for use for six weeks. Following their placebo run in, participants undertook a battery of neuropsychological tests before being randomized, and again approximately six weeks later at study completion. Primary outcome measures included neuropsychological tests from the WMS-IV, DKEFS, CVLT-II, Trails A & B and PSAT as well as measures of sleep.

## Results

Bivariate analysis indicated no significant differences between groups on any demographic variables and both groups demonstrated excellent supplement adherence (> 90%). Following the two-week placebo run in, no significant differences were found between groups on any cognitive measure. At six weeks, significant improvement was noted in tasks of delayed verbal recall and executive functioning for the Alpha BRAIN^® ^group compared to placebo (p < 0.05). Both groups demonstrated overall improvement on neuropsychological tests between time points. Analysis of variance (ANOVA) was utilized to assess the impact of randomization on neuropsychological outcome measures across time points. Results indicated significant interaction effects for improvement in delayed verbal recall for the AlphaBrain^(TM) ^group, *F *(1.61) = 4.07, p < 0.05, partial eta squared =0.06.

## Conclusions

The use of Alpha BRAIN^® ^for 6-weeks significantly improved recent verbal memory and executive function when compared with controls, in a group of healthy adults aged 18-35. Results of this trial merit further study toward the application of cognitive enhancing supplements in athletic performance.

